# Xiaochaihu decorction relieves liver fibrosis caused by *Schistosoma japonicum* infection *via* the HSP47/TGF-β pathway

**DOI:** 10.1186/s13071-020-04121-2

**Published:** 2020-05-14

**Authors:** Yuzheng Huang, Jin Lu, Yongliang Xu, Chunrong Xiong, Deshen Tong, Nannan Hu, Haitao Yang

**Affiliations:** 1 National Health Commission Key Laboratory of Parasitic Disease Control and Prevention, Jiangsu Provincial Key Laboratory on Parasite and Vector Control Technology, Jiangsu Institute of Parasitic Diseases, 117 Meiyuan Yangxiang, Wuxi, 214064 Jiangsu China; 2 Public Health Research Center, Jiangnan University, Wuxi, 214122 Jiangsu Province China; 3 Center for Global Health, School of Public Health, Nanjing Medical University, Nanjing, 211166 China

**Keywords:** Xiaochaihu decorction, *Schistosoma japonicum*, Hepatic fibrosis, HSP47, TGF-β

## Abstract

**Background:**

Hepatic fibrosis caused by chronic infection with *Schistosoma japonica* remains a serious public health problem in the world. Symptoms include inflammation, liver granuloma and fibrosis, whilst treatment options are still limited. This study aims to investigate whether and how traditional Chinese medicine Xiaochaihu decoction (XCH) could mitigate liver fibrosis caused by *S. japonicum* infection.

**Methods:**

BALB/c mice were infected with *S. japonicum* cercariae and treated with XCH for 16 weeks. Liver pathological changes were assessed by H&E and Masson staining. NIH3T3 and Raw264.7 cells were treated with *S. japonicum* egg antigens with or without XCH treatment. Quantitative real-time PCR, western blot, immunfluorescence and ELISA were performed to determine the changes of levels of fibrogenic markers.

**Results:**

XCH protected mouse liver from injuries and fibrosis caused by *S. japonicum* infection and considerably reduced egg burden in a dose-dependent manner. Infection with *S. japonicum* caused elevation of serum ALT, AST, ALP, HA and PIIINP levels and reduction of ALB and GLOB levels, which was markedly suppressed by XCH. The upregulation of TGF-β1, Hsp47, α-SMA, Col1A1 and Col3A1 in *S. japonicum-*infected mouse liver was also significantly inhibited by XCH. *Schistosoma japonicum* egg antigens promoted the expression of Hsp47, TGF-β1, Timp-1, α-SMA, Col1A1 and Col3A1 in NIH3T3 cells, and TGF-β1, CTGF, IL-13, IL-17 and IL-6 in Raw264.7 cells, which was inhibited by XCH, LY2157299 and shRNA-Hsp47.

**Conclusions:**

These results demonstrated that the hepatic protective effects of Xiaochaihu decoction were mediated by HSP47/TGF-β axis.
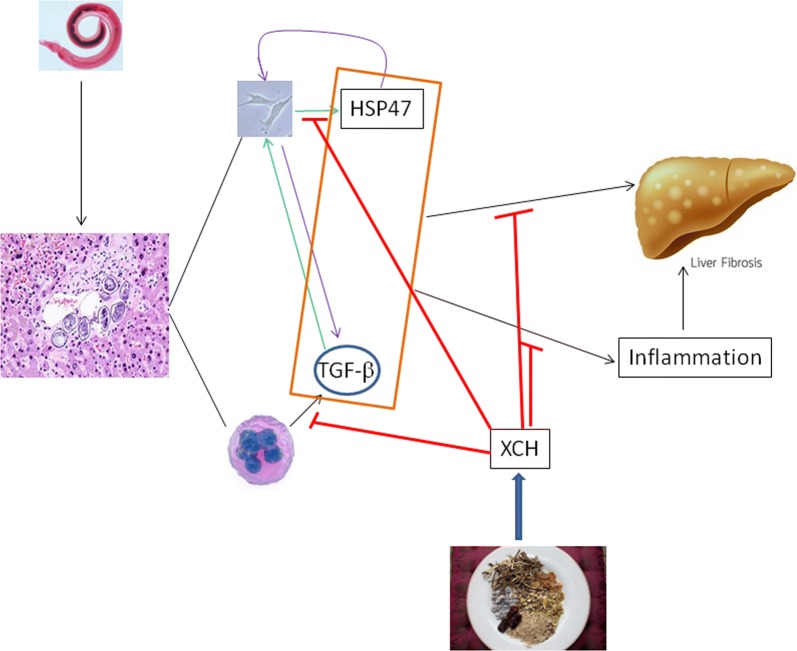

## Background

Schistosomiasis is a serious parasitic disease and caused by six species of parasites belonging to the genus *Schistosoma*, which are uniquely geographically distributed in the tropical and subtropical areas worldwide [1]. Schistosomiasis is highly debilitating with three distinct phases of clinical disease progression: acute infection; established active infection; and late chronic infection [2, 3]. Chronic and advanced schistosomiasis remain a serious public health problem in China. The marshland areas with infected snails in Anhui, Jiangxi, Hunan, and Hubei provinces are the major endemic region [4]. *Schistosoma japonicum* eggs cause granulomatous inflammation in the host liver during the acute phase and lead to chronic liver damage, which might progress to the hepatosplenic schistosomiasis with egg granuloma deposition and fibrosis on the vascular wall [5–7].

Liver cirrhosis is the advanced stage of fibrosis due to chronic inflammation [8]. Hepatic fibrosis could be induced by infectious agents such as the bacterium *Helicobacter hepaticus* [9] and the parasite *Schistosoma* [10]. Hepatic Kupffer cells are activated and bone marrow derived macrophages recruited to the liver upon exposure to inflammatory inducers or liver injuries [11]. Macrophage-produced TGF-β1 activates otherwise quiescent hepatic stellate cells which in turn secrete an extracellular matrix, leading to fibrosis [8, 11].

Heat-shock protein 47 (HSP47) is an ER-resident molecular chaperone that binds specifically to procollagen [12]. The expression pattern of HSP47 closely correlates with collagen expression [13]. Mice with whole body Hsp47 knockout (KO) die after 11.5 days *post-coitus* (dpc) due to almost complete loss of the mature type I collagen and fibril structures of type I collagen in embryonic mesenchymal tissues [14]. Chondrocyte-specific Hsp47 KO mice die around birth with severe generalized chondrodysplasia and bony deformities due to reduced levels of type II and type XI collagen [15]. HSP47 has been shown to regulate the biosynthesis, processing, transport, secretion and assembly of collagens [16, 17]. Thus, Hsp47 is used as a target for treating collagen-related diseases including skin and lung fibrosis [18, 19].

Xiaochaihu decorction (XCH) was first described in the *Shang Han Lun* to treat febrile diseases by the physician Zhong-Jing Zhang around AD200, including Radix Bupleuri (Chinese thorowax root), Radix Scutellariae (huangqin or baical skullcap root), Rhizoma Pinelliae (banxia or pinellia tuber), Radix Ginseng (renshen or ginseng), Radix Glycyrrhizae (gancao or licorice root), Rhizoma Zingiberis Recens (shengjiang or fresh ginger) and Fructus Jujubae (dazhao or Chinese date) [20]. XCH has been shown to protect against experimental liver injuries [21, 22], prevent and treat experimental hepatic and pancreatic fibrosis [23–25]. This study aims to investigate the effects of XCH on hepatic fibrosis of *S. japonicum* infected mice and the underlying molecular mechanism.

## Methods

### Cell culture and treatment

NIH 3T3 cells and Raw264.7 cells were obtained from the Cell Bank of Shanghai Institute of Biochemistry and Cell Biology, Chinese Academy of Sciences (Shanghai, China). The cells were maintained in DMEM medium containing 10% FBS and incubated at 37 °C with 5% humidified CO_2_. NIH3T3 were treated with XCH, LY2157299, TGF-β1 and shRNA-HSP47 for 48 h. Raw264.7 cells were treated with XCH and LY2157299 for 48 h.

*Schistosoma japonicum* egg antigen (0.01 g/ml in phosphate-buffered saline) was obtained from the Jiangsu Institute of Parasitic Diseases (Wuxi, China) and diluted to the working concentration (10 μg/ml) in DMEM containing 2% FBS immediately before use.

### Western blotting

The western blotting procedure was performed as described in our previous reports [26]. Briefly, after required treatments, the total protein of the cell samples was isolated using radioimmunoprecipitation assay (RIPA) lysis buffer. Equal amounts of total protein were separated by appropriate SDS-PAGE and transferred to a polyvinylidene fluoride (PVDF) membrane. After blocking with skimmed milk, the PVDF membrane was incubated with specific primary antibodies followed by incubation with the corresponding secondary antibodies. Protein bands were detected using the Bio-Rad ChemiDocTM (Hercules, CA, USA). β-actin was used as an internal control.

### Real-time quantitative polymerase chain reaction (RT-qPCR) analysis

NIH3T3 were treated with XCH, LY2157299, TGF-β1 and shRNA-HSP47. Raw 264.7 cells were treated with XCH and LY2157299. Total RNA was subsequently extracted from cells using RNAiso plus (Takara, Dalian, China) according to the manufacturer’s instructions. The total RNA was reverse-transcribed into cDNA using an M-MLV Reverse Transcriptase kit (Invitrogen, Shanghai, China), and the resultant cDNA mixture was diluted 10-fold in RNase-free ddH_2_O. RT-qPCR amplification was performed using SYBR Premix Ex Taq^TM^ kit (Takara) in a 20 μl reaction containing 0.4 μl of each primer, 0.4 μl SYBR Green Dye and 2 μl of cDNA. The PCR primers are listed in Additional file 1: Table S1. RT-qPCR was carried out on a Roche LightCycler 480II (Roche Diagnostics, Shanghai, China) using the following program: 95 °C for 30 s; followed by 40 cycles of 95 °C for 5 s and 58 °C for 34 s. The relative gene expression level was calculated with 2^-ΔΔCq^ method using β-actin as an internal control.

### Immunofluorescence staining

The localization of COL3Al and COL1Al were analyzed by immunofluorescence staining. Cells were fixed with 4% formaldehyde in PBS containing 1% sucrose at 37 °C for 15 min. The fixative was then removed and the samples were permeabilized with 0.1% of Triton-X 100 at 4 °C for 10 min. This was followed by a blocking step with 1% bovine serum albumin (BSA)/PBS at 37 °C for 5 min. After blocking, the cells were incubated with anti-COL3Al (Cat# ab7778, 1:200 dilution; Abcam, Cambridge, MA, USA) or COL1Al (Cat# ab34710, 1:500 dilution; Abcam) in 1% BSA/PBS for 1 h at 37 °C. Cells were then incubated with a Cy-3 conjugated secondary goat anti-rabbit antibody (Jackson ImmunoResearch, West Grove, PA, USA) for 30 min at 4 °C. The samples were mounted in DAPI to stain the nuclei. Images of the stained substrates were taken *via* a Leica IX71 fluorescent microscope (Leica Microsystems GmbH, Wetzlar, Germany).

### ELISA assay

Raw264.7 cells (1 × 10^4^ cells/well) were plated in 96-well plates for 24 h and then incubated with or without *S. japonicum* egg antigens for 48 h. The culture media were collected for the CTGF, TGF-β1, IL-13, IL-17 and IL-6 assays. The concentrations of CTGF, TGF-β1, IL-13, IL-17 and IL-6 in the cell culture media were determined by commercial ELISA kits (RJ17277, RJ17947, RJ16937, RJ16942, RJ16958 respectively, Renjie Bio (http://www.rjkit.com/products.html), Shanghai, China) according to the manufacturer’s instructions.

### Parasites and animals

Cercariae of *S. japonicum* (Chinese strain) were obtained from infected *Oncomelania hupensis* snails fed at the Vector Biology Laboratory in our institute using a standard procedure [27]. Sixty healthy adult female BALB/c mice with body weights of *c.*20 g were purchased from Yangzhou University.

Mice were randomly assigned into two groups, a control group and an infected group. Infection of mice with 15 *S. japonicum* cercariae was carried out through shaved abdominal skin for 30 min and no other treatment or manipulation of the animals was involved. Using the same procedure, the control mice were exposed to physiological saline. Then the uninfected mice were divided into two groups (10 mice in each group) to receive saline or Xiaochaihu decoction (XCH-H). The infected mice were divided into four groups (10 mice in each group) to receive saline or one of three doses (XCH-L, XCH-M and XCH-H) of Xiaochaihu decoction by gavage, respectively.

### Xiaochaihu decoction

XCH was made from 24 g bupleurum, 9 g astragalus, 9 g ginseng, 9 g pinellia, 9 g licorice, 9 g ginger root and 40 g jujube, which was boiled in 500 ml H_2_O and simmered until the volume reduced to 100 ml. The dosage of XCH per mouse was as follows: XCH-L (5 ml/kg/day); XCH-M (15 ml/kg/day); and XCH-L (30 ml/kg/day). The final concentration for cell culture was 1.67 μl/ml.

### Identification of infected mice

Stool samples were also collected every week after infection and examined using kato-katz thick smears [27]. Infected with *S. japonicum* mice were successfully identified with parasite eggs found in the feces of all mice at 6 weeks post-infection. The liver was extracted from all mice 16 weeks post-infection. Hematoxylin and eosin (H&E) and Masson staining of liver tissue was performed to assess pathological changes.

### Liver function and fibrosis biomarkers

Mouse serum alanine aminotransferase (AST), aspartate aminotransferase (ALT), and alkaline phosphatase (ALP) activities were measured using specific assay kits (ab105134 for AST, ab105135 for ALT, ab83369 for AP, Abcam, Shanghai, China). Serum hyaluronic acid (HA), N-terminal propeptide of collagen III (PIIINP), albumin (ALB), and globulin (GLOB) levels were analyzed with commercial ELISA kits (CSB-E08121m for HA, CUSABIO, Houston, TX, USA; LS-F25207 for PIIINP, LSBio, Seattle, WA; ab108791 for albumin, and ab151276 for globulin, Abcam, Shanghai, China).

### Statistical analysis

Results are expressed as the mean ± standard deviation. Statistical analyses were performed with Graphpad Prism 6 (Graphpad, San Diego, CA, USA). The differences among treatment groups were analyzed by a one-way ANOVA followed by Tukey’s *post-hoc* test. A *P*-value less than 0.05 was considered statistically significant.

## Results

### XCH alleviated liver granuloma and fibrosis in mice caused by *S. japonicum*

**Fig. 1:**
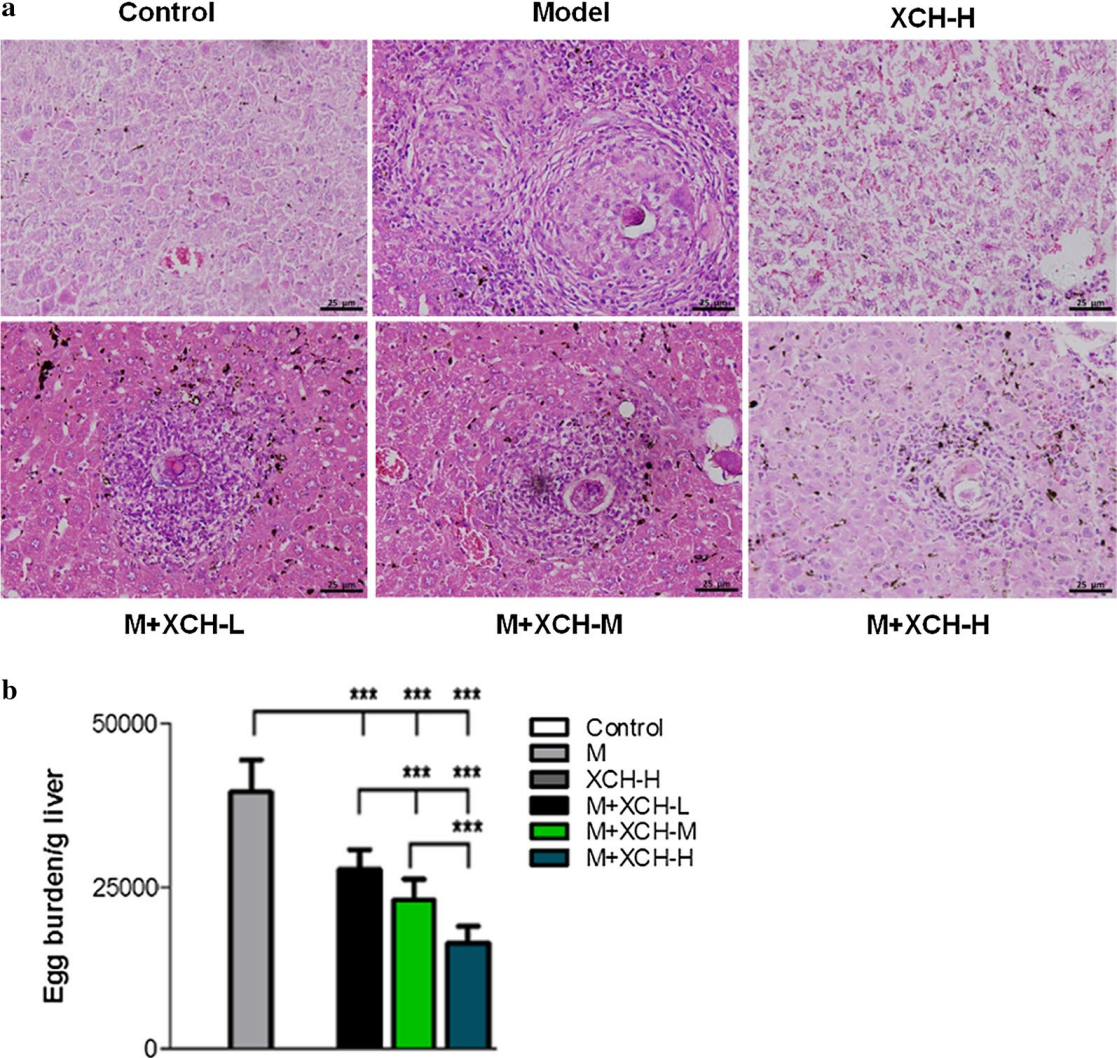
Xiaochaihu (XCH) decoction reduced *Schistosoma japonicum* burden of mouse liver. BALB/c mice were infected with 15 *S. japonicum* cercariae and treated with either saline or Xiaochaihu decoction. Mice were sacrificed and liver tissues were collected 16 weeks post-infection. **a** H&E staining showing the pathological changes and *S. japonicum* eggs in mouse liver. **b** Statistical differences in egg burden of infected mice. *Abbreviations*: M, Model, *S. japonicum* infection; XCH-L (M, H), Xiaochaihu decoction low (middle, high) dose. *Scale-bars*: **a**, 25 µm. ***P < 0.0001

**Fig. 2:**
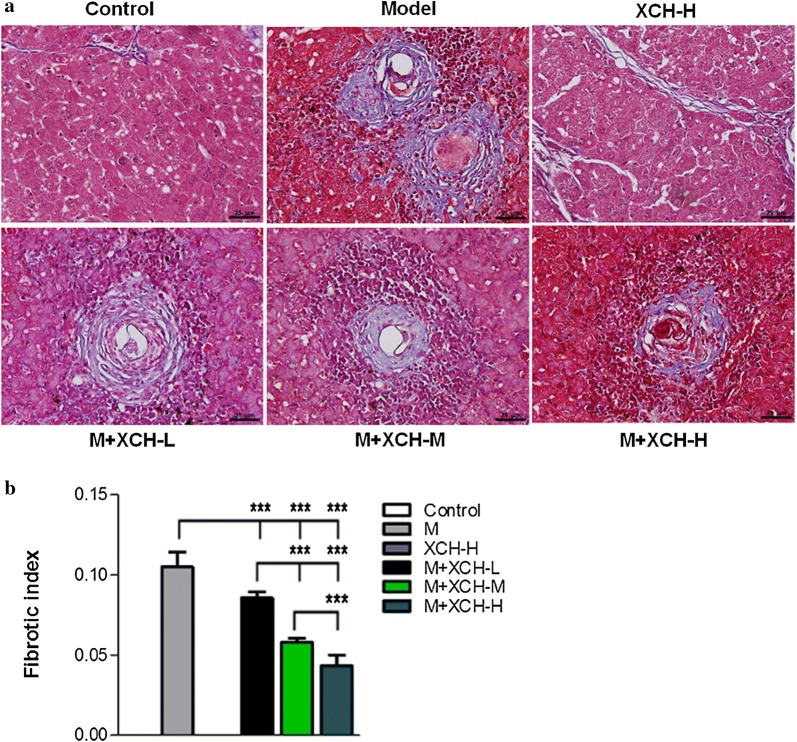
Xiaochaihu (XCH) decoction reduced *Schistosoma japonicum* caused liver fibrosis in mice. BALB/c mice were infected with 15 *S. japonicum* cercariae and treated with either saline or Xiaochaihu decoction. Mice were sacrificed and liver tissues were collected 16 weeks post-infection. **a** Masson staining showing the amount of fibrotic tissue in mouse liver. **b** Statistical differences in egg burden of infected mice. *Abbreviations*: M, Model, *S. japonicum* infection; XCH-L (M, H), Xiaochaihu decoction low (middle, high) dose. *Scale-bars*: **a**, 25 µm. ***P < 0.0001

*Schistosoma japonicum* infection caused destruction of the histological structure of mouse liver with a large number of *S. japonicum* eggs present around the portal veins (Fig. 1a, b). Meanwhile, *S. japonicum-*infected mouse liver significantly increased fibrotic tissue compared to uninfected healthy mice (Fig. 2). Xiaochaihu decoction treatment markedly reduced the amount of *S. japonicum* eggs (ANOVA: *F*_(5, 54)_ = 115.2, *P* < 0.001) (Fig. 1), the size of single liver granuloma and fibrotic tissue (ANOVA: *F*_(5,54)_ = 111.4, *P* < 0.001) (Fig. 2) in infected mouse livers in a dose-dependent manner.

### XCH improved hepatic dysfunction and fibrosis caused by *S. japonicum*

*Schistosoma japonicum* infection resulted in a drastic increase in serum activities of alanine aminotransferase (ALT), aspartate aminotransferase (AST), alkaline phosphatase (ALP) as well as hyaluronic acid (HA) and N-terminal propeptide of collagen III (PIIINP) levels and decreases of albumin (ALB) and globulin (GLOB) levels (Table 1). Xiaochaihu decoction significantly decreased ALT, AST and ALP activities and HA and PIIINP levels (decrease range of 22–57%) while increased levels of ALB (51%) and GLOB (27%) were detected in mice infected with *S. japonicum*. (Table 1).

**Table 1:** Parameters of hepatic function and fibrosis

	Control	SJ	SJ+XCH
ALT (U/l)	22.7 ± 4.7	86.3 ± 11.0^*^	40.7 ± 7.8^*#^
AST (U/l)	116.7 ± 15.3	169.0 ± 14.0^*^	132.0 ± 15.9^*#^
ALP (U/l)	31.3 ± 0.6	73.3 ± 4.7^*^	42.7 ± 3.1^*#^
ALB (g/l)	42.5 ± 2.3	23.3 ± 2.8^*^	35.1 ± 0.8^*#^
GLOB (g/l)	50.8 ± 3.0	33.5 ± 4.2^*^	42.7 ± 2.6^*#^
ALB/GLOB	0.8	0.7	0.8
HA (g/l)	411.7 ± 31.8	1924.3 ± 179.8^*^	1168.7 ± 155.9^*#^
PIIINP (g/l)	1.9 ± 0.1	3.7 ± 0.3^*^	2.2 ± 0.2^#^

*Note*: Data are shown as mean ± SD

^*^P < 0.05 compared to control, ^#^*P* < 0.05 compared to SJ

*Abbreviations*: SJ, *Schistosoma japonicum*; XCH, Xiaochaihu decoction; ALB, albumin; ALP, alkaline phosphatase, ALT, alanine aminotransferase; AST, aspartate aminotransferase; GLOB, globulin, HA, hyaluronic acid; PIIINP, N-terminal propeptide of collagen III

### XCH inhibited upregulation of hepatic fibrogenic genes caused by infection with *S. japonicum*

**Fig. 3:**
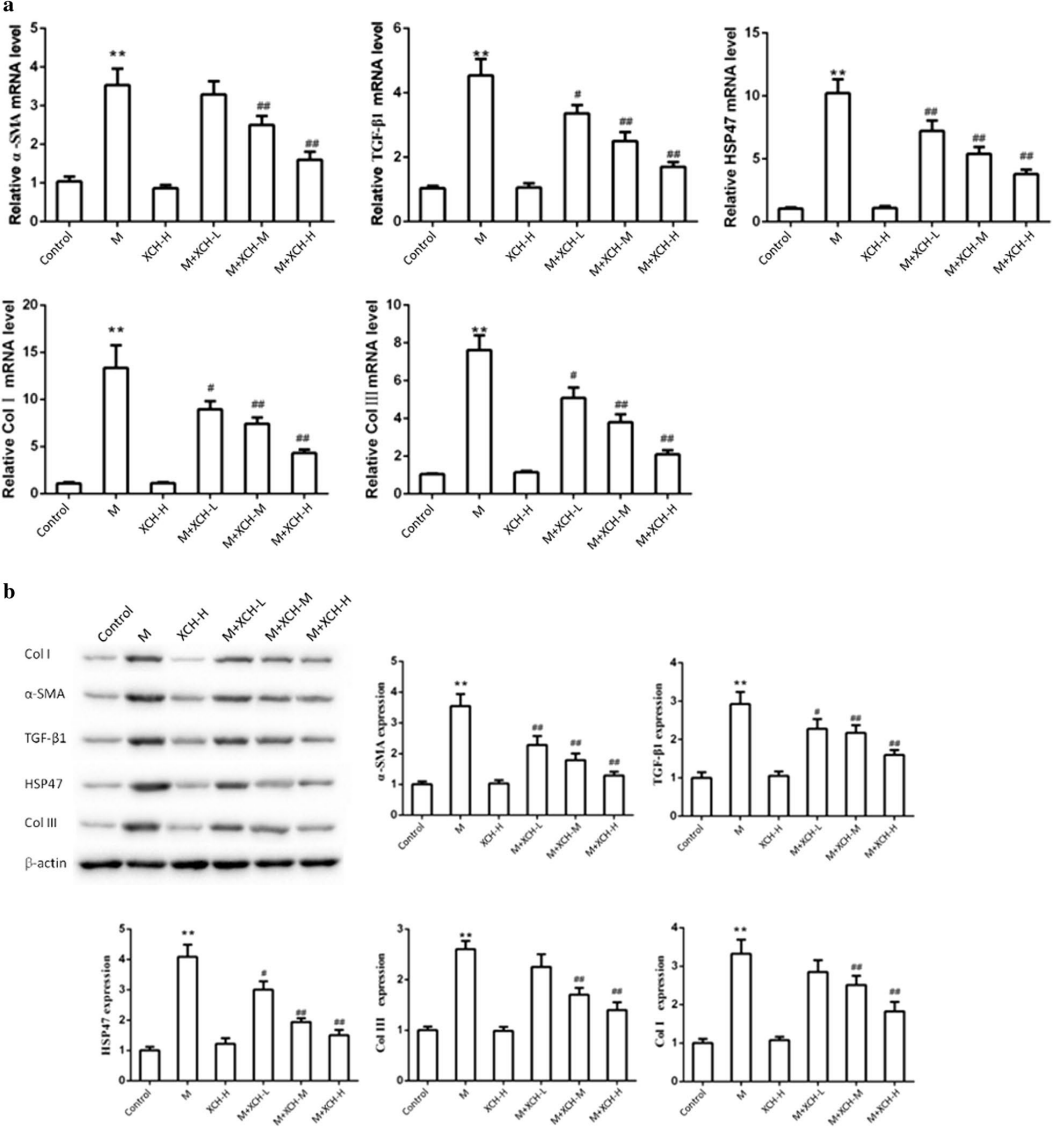
Xiaochaihu (XCH) decoction inhibited *Schistosoma japonicum* induced upregulation of hepatic fibrogenic genes. Total RNA and protein were extracted from mouse livers after *S. japonicum* infection and treatment with Xiaochaihu decoction. **a** The changes of TGF-β1, HSP47, α-SMA, Col III and Col I mRNA levels of *S. japonicum-*infected mouse liver were assessed by quantitative real-time PCR. **b** Hepatic protein levels of Col I, α-SMA, TGF-β1, HSP47 and Col III were assessed by western blot. *Abbreviations*: M, Model, *S. japonicum* infection; XCH-L (M, H), Xiaochaihu decoction low (middle, high) dose. *P* < 0.05, **P < 0.01

As XCH relieved hepatic pathological changes caused by infection with *S. japonicum* including liver granuloma and fibrosis, we next assessed the expression levels of genes related to hepatic fibrosis. *Schistosoma japonicum* infection caused substantial upregulation of hepatic TGF-β1, Hsp47, α-SMA, Col1A1 and Col3A1 at both mRNA (Fig. 3a) and protein (Fig. 3b) levels. Xiaochaihu decoction dose-dependently inhibited the overexpression of these fibrotic genes in *S. japonicum-*infected mouse livers (ANOVA: α-SMA, *F*_(5, 54)_ = 67.3, *P* = 0.0012; Col1A1, *F*_(5, 54)_ = 252.2, *P* < 0.0001; HSP47, *F*_(5, 54)_ = 73.3, *P* = 0.0010; COL3A1, *F*_(5, 54)_ = 173.4, *P* = 0.0002; TGF-β1, *F*_(5, 54)_ = 70.9, *P* = 0.0011) (Fig. 3).

### XCH inhibited fibroblast activation and collagen production through HSP47 and TGF-β

**Fig. 4:**
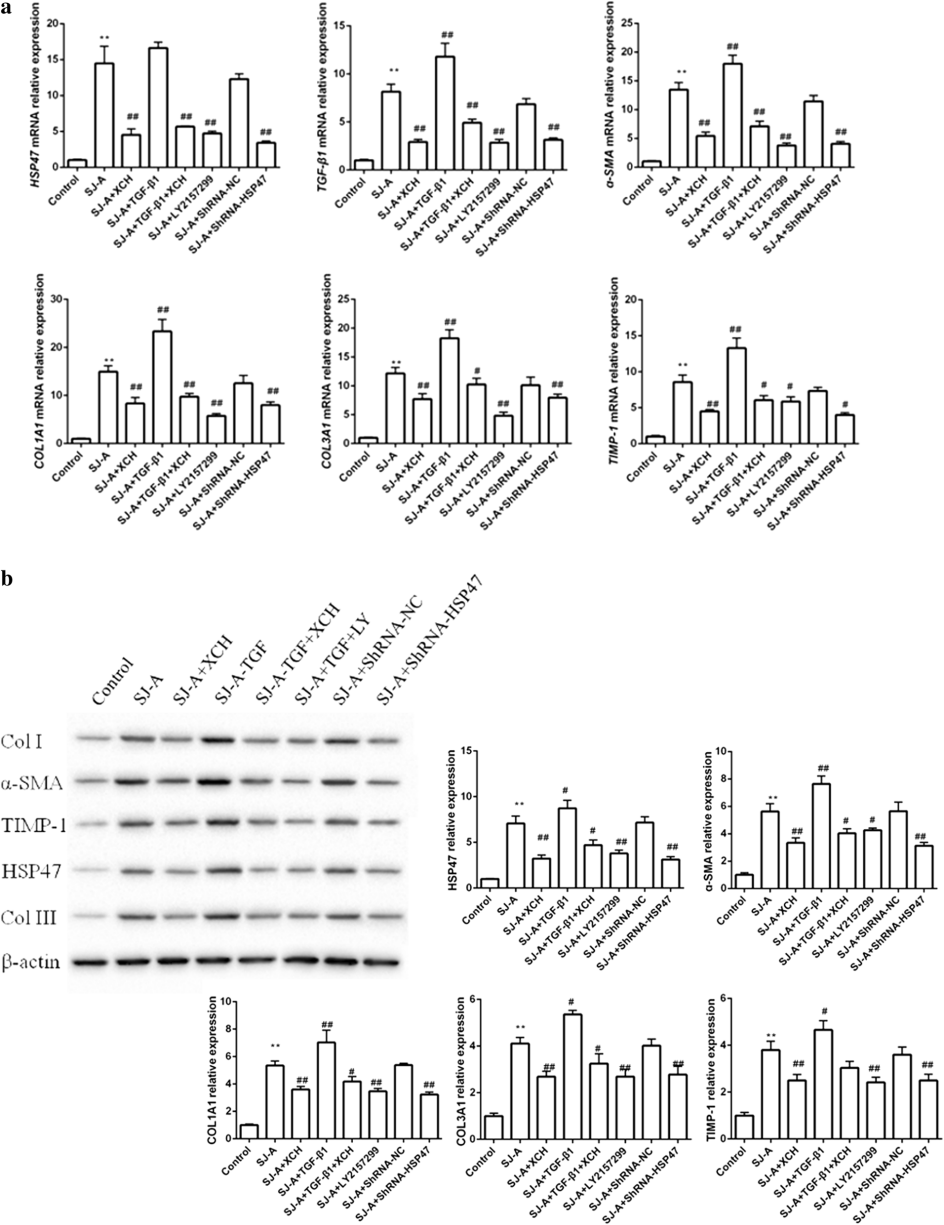
Xiaochaihu (XCH) decoction inhibited *Schistosoma japonicum* induced fibroblast activation. **a** Quantitative real-time PCR showed that *S. japonicum* egg antigens induced upregulation of HSP47, TGF-β1, Timp-1, α-SMA, Col1A1 and Col3A1 in NIH3T3 fibroblasts, which was inhibited by XCH, TGF-β receptor inhibitor LY2157299, and shRNA targeting Hsp47. **b** The changes of α-SMA, HSP47, Timp-1, Col 1 and Col 3 protein level in NIH3T3 fibroblasts under specified treatments were evaluated by western blot. *Abbreviations*: SJ-A, *S. japonicum* egg antigens; LY, LY2157299. *P < 0.05, **P < 0.01 compared to the control, #*P* < 0.05, ##*P* < 0.01 compared to *S. japonicum* egg antigens

**Fig. 5:**
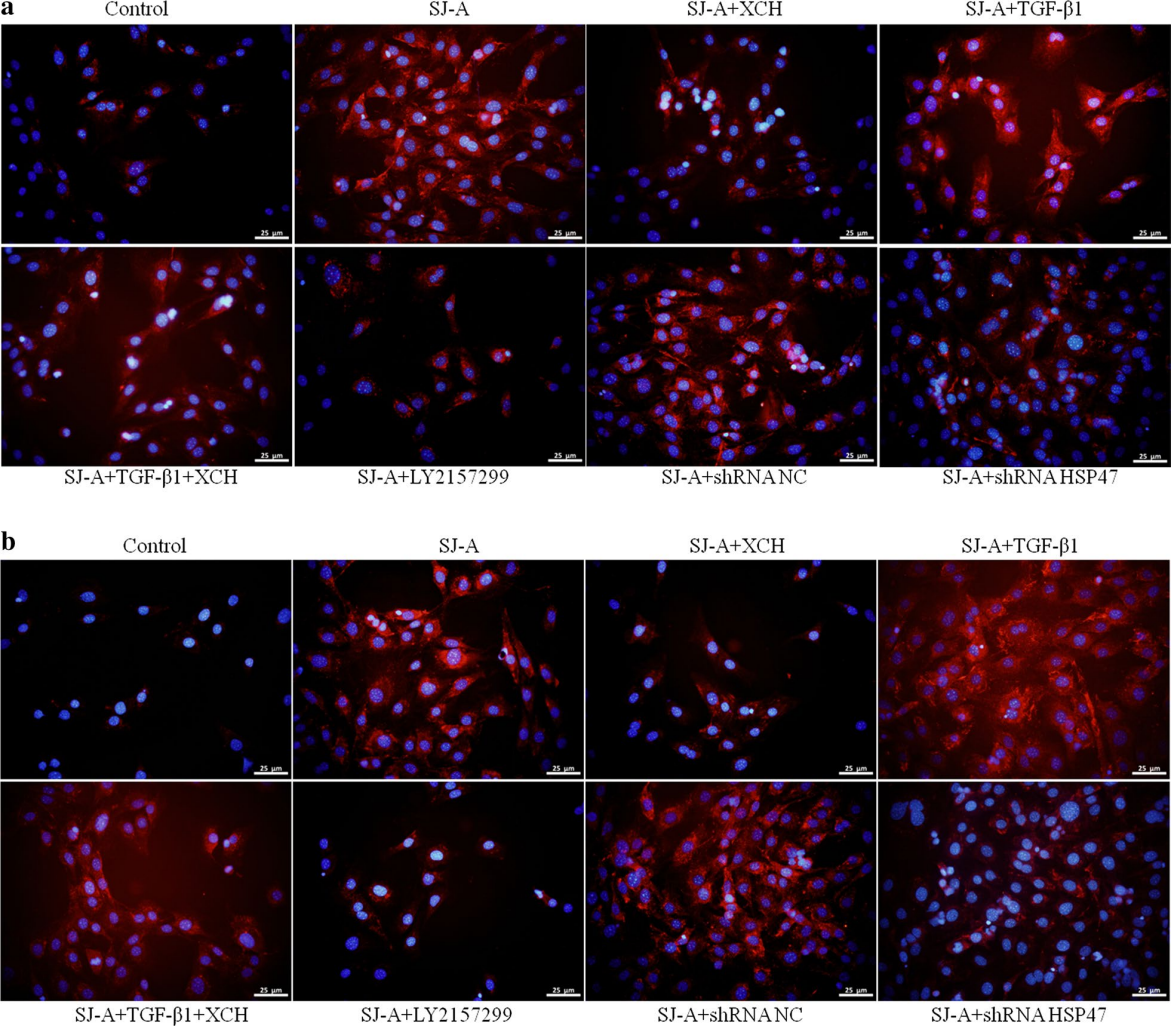
Xiaochaihu (XCH) decoction reduced *Schistosoma japonicum* induced extracellular matrix deposition. The production and deposition of collagen 1 (**a**) and 3 (**b**) were assayed by immunofluorescence. *Abbreviations*: SJ-A, *S. japonicum* egg antigens; LY, LY2157299. *Scale-bars*: 25 µm

To elucidate the mechanism governing the inhibition of XCH on *S. japonicum* infection caused hepatic fibrosis, we treated NIH3T3 mouse fibroblast with *S. japonicum* egg antigens in the presence or absence of TGF-β or shRNA targeting Hsp47 (shRNA-Hsp47). *Schistosoma japonicum* egg antigens strongly stimulated the expression of Hsp47, TGF-β1, Timp-1, α-SMA, Col1A1 and Col3A1 in NIH3T3 cells, which was significantly inhibited by XCH, TGF-β receptor 1 (TGF-βR1) inhibitor LY2157299 and shRNA-Hsp47 (ANOVA: mRNA: ɑ-SMA, *F*_(7, 16)_ = 533.7, *P* < 0.0001; Col1A1, *F*_(7, 16)_ = 525.2, *P* < 0.0001; HSP47; *F*_(7, 16)_ = 127.9, *P* = 0.0003, COL3A1, *F*_(7, 16)_ = 443.7, *P* < 0.0001; TGF-β1, *F*_(7, 16)_ = 1701.7, *P* < 0.0001; TIMP-1, *F*_(7, 16)_ = 86.9, *P* = 0.0007. Protein: ɑ-SMA, *F*_(7, 16)_ = 147.4, *P* < 0.0001; Col I, *F*_(7, 16)_ = 285.3, *P* < 0.0001; HSP47, *F*_(7, 16)_ = 15528.6, *P* < 0.0001; COL III, *F*_(7, 16)_ = 465.2, *P* < 0.0001; TIMP-1, *F*_(7, 16)_ = 129.8, *P* = 0.0003) (Fig. 4a, b). Exogenous TGF-β1 further augmented the upregulation of fibrotic genes by *S. japonicum* egg antigens, which was markedly suppressed by XCH (Fig. 4a, b). Moreover, the deposition of collagen 1 (Fig. 5a) and collagen 3 (Fig. 5b) was also drastically stimulated by *S. japonicum* egg antigens and further increased by TGF-β1, which was inhibited by XCH and shRNA-Hsp47.

### XCH inhibited the production of fibrogenetic cytokines induced by *S. japonicum* egg antigens in NIH3T3 cells

**Fig. 6:**
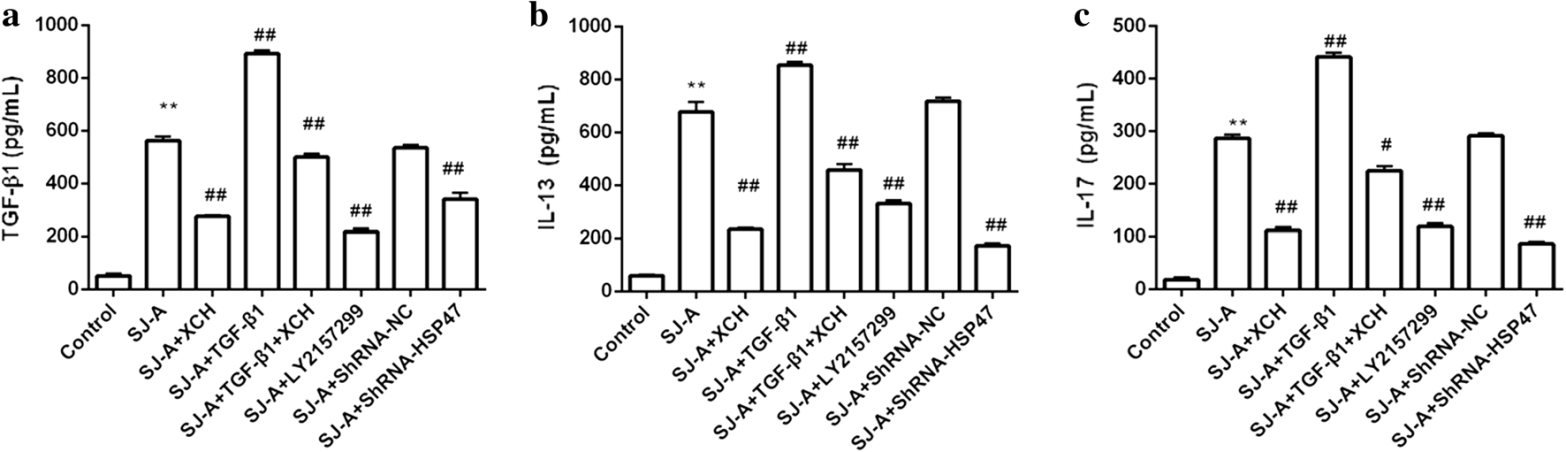
Xiaochaihu (XCH) decoction inhibited *Schistosoma japonicum* induced fibrogenic cytokine production by fibroblasts. NIH3T3 cells were treated with *S. japonicum* egg antigens in the presence or absence of Xiaochaihu decoction, LY2157299 and shRNA-HSP47. The medium levels of TGF-β (**a**), IL-13 (**b**), and IL-17 (**c**) of NIH3T3 cells were measured by ELISA. *Abbreviations*: SJ-A, *S. japonicum* egg antigens. **P < 0.01 compared to control, #*P* < 0.05 compared to infection, ##*P* < 0.01 compared to infection

*Schistosoma japonicum* egg antigens strongly stimulated the excretion of TGF-β1 (Fig. 6a), interleukin 13 (IL-13, Fig. 6b), and IL-17 (Fig. 6c) by NIH3T3 fibroblasts. Treatment with XCH, TGF-βR1 antagonist LY2157299, and shRNA-Hsp47 significantly reduced medium TGF-β1 (ANOVA: *F*_(7, 16)_ = 2520.5, *P* < 0.0001) (Fig. 6a), IL-13 (ANOVA: *F*_(7, 16)_ = 774.8, *P* < 0.0001) (Fig. 6b), and IL-17 (ANOVA: *F*_(7, 16)_ = 3481.2, *P* < 0.0001) (Fig. 6c) levels of NIH3T3 cells exposed to *S. japonicum* egg antigens.

### XCH inhibited *S. japonicum-*induced activation of macrophages

**Fig. 7:**
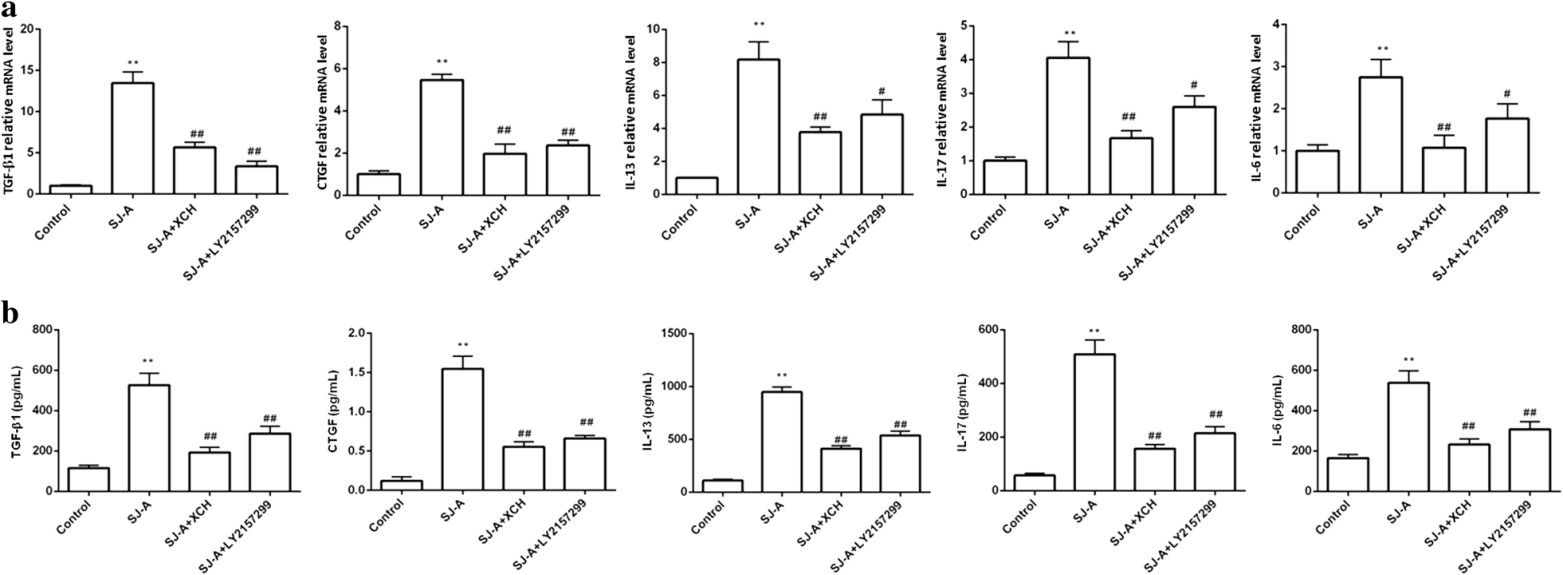
*Schistosoma japonicum* induced activation of macrophages was inhibited by Xiaochaihu decoction (XCH). RAW264.7 cells were treated with *S. japonicum* egg antigens in the presence or absence of Xiaochaihu decoction and LY2157299. **a** The mRNA levels of TGF-β1, CTGF, IL-13, IL-17 and IL-6 of Raw264.7 macrophages were analyzed by quantitative real-time PCR. **b** The levels of medium TGF-β1, CTGF, IL-13, IL-17 and IL-6 of Raw264.7 macrophages were assessed by ELISA. *Abbreviations*: SJ-A, *S. japonicum* egg antigens. **P < 0.01 compared to control, #*P* < 0.05 compared to infection, ##*P* < 0.01 compared to infection

To investigate the effects of *S. japonicum* infection and Xiaochaihu decoction on the activation of macrophages, we treated Raw264.7 mouse macrophages with *S. japonicum* egg antigens in the presence or absence of XCH and LY2157299. *Schistosoma japonicum* egg antigens markedly increased the mRNA levels of TGF-β1, CTGF, IL-13, IL-17 and IL-6, and the increases were significantly inhibited by either XCH or LY2157299 (ANOVA: TGF-β1, *F*_(3, 8)_ = 1479.0, *P* < 0.0001, CTGF, *F*_(3, 8)_ = 973.8, *P* < 0.0001; IL-13, *F*_(3, 8)_ = 5157.7, *P* < 0.0001; IL-17, *F*_(3, 8)_ = 33332.3, *P* < 0.0001; IL-6, *F*_(3, 8)_ = 2839.5, *P* < 0.0001) (Fig. 7a). Medium levels of TGF-β1, CTGF, IL-13, IL-17, and IL-6 of Raw264.7 macrophages were drastically increased by *S. japonicum* egg antigens (Fig. 7b). XCH and LY2157299 treatment reduced medium TGF-β1, CTGF, IL-13, IL-17, and IL-6 levels by 40–60% of *S. japonicum* egg antigens-treated Raw264.7 macrophages (ANOVA: TGF-β1, *F*_(3, 8)_ = 244.4, *P* < 0.0001; CTGF, *F*_(3, 8)_ = 472.8, *P* < 0.0001; IL-13, *F*_(3, 8)_ = 125.8, *P* = 0.0004; IL-17, *F*_(3, 8)_ = 100.3, *P* = 0.0006; IL-6, *F*_(3, 8)_ = 35.7, *P* = 0.0039) (Fig. 7b).

## Discussion

The traditional Chinese medicine Xiaochaihu decoction showed a strong curative effect against *S. japonicum-*induced hepatic fibrosis *via* a Hsp47/TGF-β1 axis. XCH reduced *S. japonicum* egg burden and fibrotic tissues in infected mouse livers, inhibited hepatic expression of fibrogenic genes TGF-β1, Hsp47, α-SMA, Col1A1 and Col3A1, upregulated by *S. japonicum* infection. *Schistosoma japonicum* caused changes in ALT, AST, ALP, ALB, GLOB, HA and PIIINP levels, which were considerably reversed by XCH. *Schistosoma japonicum* egg antigens induced the expression of fibrogenic genes Hsp47, TGF-β1, Timp-1, α-SMA, Col1A1 and Col3A1 in NIH3T3 mouse fibroblasts, which was inhibited by XCH and shRNA-Hsp47. Fibrogenic cytokines induced by *S. japonicum* egg antigens were inhibited by XCH, LY2157299 and shRNA-Hsp47 in NIH3T3 fibroblasts and by XCH and LY2157299 in Raw264.7 macrophages.

Hepatic fibrosis is the most severe clinical consequence of *S. japonicum* infection [5, 28] so a great effort has been made to look for reagents protecting against liver fibrosis of infected individuals. Polyinosinic-polycytidylic acid (poly I:C) treatment reduced collagen deposition and stellate cell activation in *S. japonicum-*infected mouse liver accompanied by upregulation of Th1 cytokines interferons α, β and γ, TNF-α, IL-10, and IL-12 and downregulation of Th2 cytokines IL-4 and IL-5 [29]. Boswellic acid-containing extracts of the oleogum resin from *Boswellia serrata* considerably reduced the size of liver granuloma and serum levels of ALT and AST but did not impact the egg burden in *S. japonicum-*infected mice [30]. Corilagin improved the health and reduced egg burden in *S. japonicum-*infected mice through blocking TGF-β signaling by upregulating SMAD7 and inhibiting the activation of SMAD1/2 and ERK1/2 [31]. Genistein protected against liver fibrosis caused by *S. japonicum* and decreased the extent of hepatic granuloma *via* inhibiting activation of NF-κB signaling pathway and led to downregulation of inflammatory cytokines MCP1, TNFα, IL1β, IL4, IL10 in infected mice [32]. These data indicate that some plant-derived compounds are effective in treating hepatic fibrosis caused by *S. japonicum* infection. The present study demonstrated the efficacy of Xiaochaihu decoction in reducing egg burden and liver fibrosis in *S. japonicum* infected mice.

**Fig. 8:**
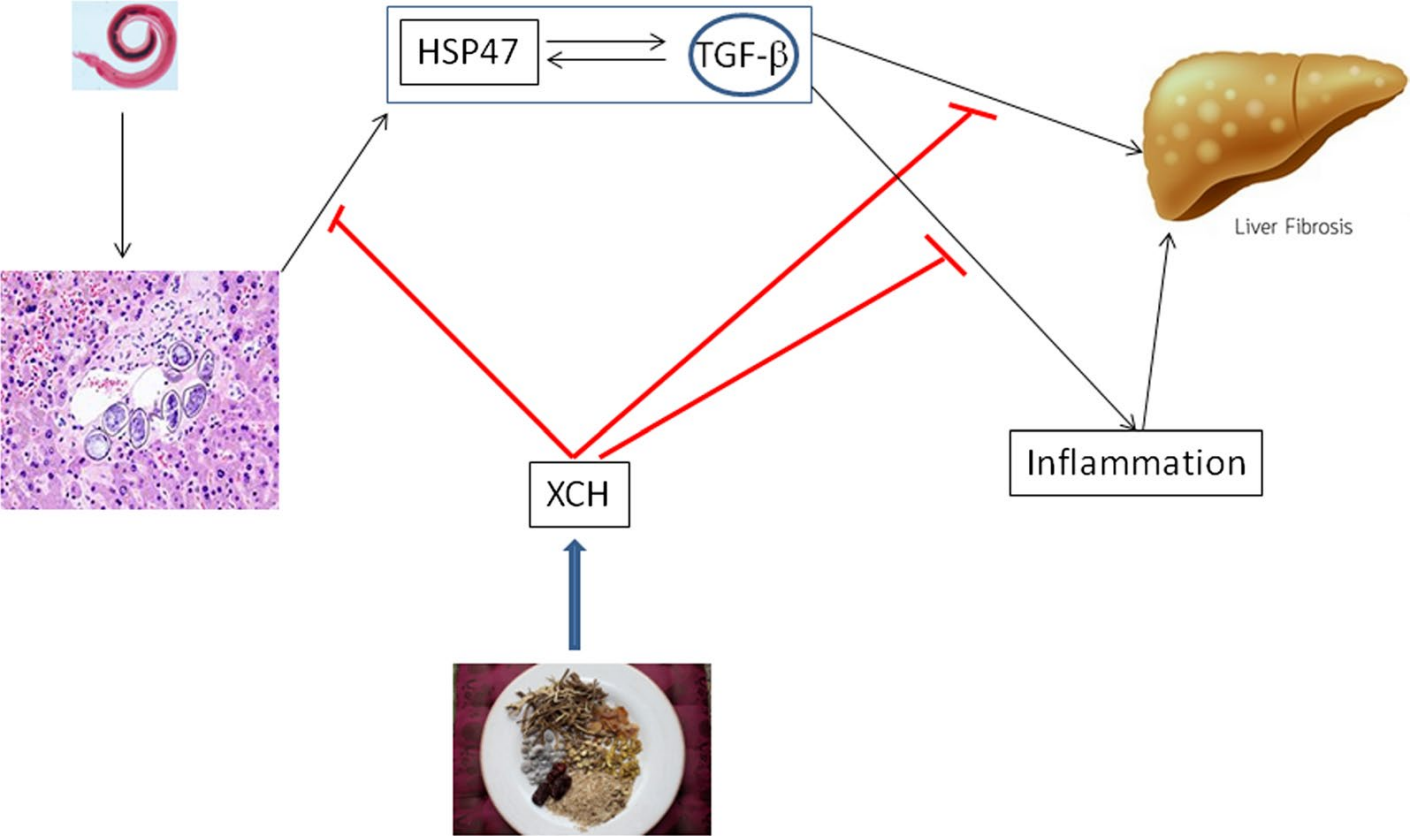
A proposed model for explaining how XCH alleviates *S. japonicum* infection induced liver fibrosis. XCH inhibits *S. japonicum* infection, survival, and proliferation in mouse liver, depresses *S. japonicum* infection induced upregulation of HSP47 and TGF-β production, and inhibits *S. japonicum-*caused inflammation, which in turn blocks the activation of hepatic stellate cells and ECM deposition

Collagen-specific chaperon HSP47 has been shown to be involved in collagen-related diseases. HSP47 was found to be overexpressed in skin fibroblasts and peripheral blood mononuclear cells from scleroderma patients. Silencing HSP47 blocked TGF-β induced collagen overproduction [33]. HSP47 was overexpressed in glioblastoma multiforme and promoted a stem-like property of primary glioma cells and the upregulation of extracellular matrix genes, which was inhibited by blocking the TGF-β pathway [34]. Chronic graft-*versus*-host disease after allogeneic hematopoietic stem cell transplantation in mice showed massive skin fibrosis with elevated collagen deposition and F4/80+ macrophage infiltration, which was alleviated by HSP47 small interfering RNA delivered by vitamin A-coupled liposomes [16]. HSP47 was upregulated by *S. japonicum* infection and short hairpin (sh)RNA targeting Hsp47 markedly reduced collagen deposition in NIH3T3 cells and *S. japonicum*-infected mouse livers. The mouse survival rate was prolonged by shRNA-Hsp47 in a dose-dependent manner [35]. HSP47 was recently shown to modulate the activation of hepatic stellate cells by regulating the expression of endothelin receptor A and endothelin receptor B in *S. japonicum-*infected mice [36]. The current study demonstrated that XCH inhibited inflammatory responses and TGF-β excretion of macrophages and the activation and ECM secretion of fibroblasts through inhabiting the expression of Hsp47 (Fig. 8).

## Conclusions

The present data show that XCH potently inhibited the expression of Hsp47 of both *S. japonicum-*infected mouse liver and NIH3T3 cells, which led to downregulation of other fibrogenic genes IL-13, IL-17, TGF-β1, Timp-1, α-SMA, Col1A1 and Col3A1. The remarkably decreased levels of fibrogenic cytokines, signaling proteins and extracellular matrix-related proteins resulted in a significant relieve of hepatic fibrosis induced by *S. japonicum*. These results indicate that Xiaochaihu decoction might be an effective therapeutic option for liver fibrosis caused by *S. japonicum* infection.

## Supplementary information

**Additional file 1: Table S1.** Sequence information for primers used in the study.

## Data Availability

Data supporting the conclusions of this article are included within the article and its additional file.

## Abbreviations

XCH: Xiaochaihu decoction
PCR: polymerase chain reaction
ELISA: enzyme-linked immunosorbent assay
ALT: alanine aminotransferase
AST: aspartate aminotransferase
ALP: alkaline phosphatase
HA: hyaluronic acid
PIIINP: amino-terminal pro-peptide of type III pro-collagen
ALB: albumin
GLOB: globulin
TGF-β1: transforming growth factor beta 1
Hsp47: heat shock protein 47
α-SMA: alpha smooth muscle actin
Col1A1: collagen type I alpha 1 chain
Col3A1: collagen type III alpha 1 chain
CTGF: connective tissue growth factor
IL: interleukin
dpc: days *post-coitus*
KO: knockout
PVDF: polyvinylidene fluoride
RT-qPCR: real-time quantitative polymerase chain reaction
