# Supplementary material for: Xiaochaihu decorction relieves liver fibrosis caused by Schistosoma japonicum infection via the HSP47/TGF-β pathway
Source: Parasit Vectors. 2020 May 14;13:254. doi: 10.1186/s13071-020-04121-2 (PMC7227055; doi:10.1186/s13071-020-04121-2)
Supplement: Supplementary file 1 — Additional file 1: Table S1. Sequence information for primers used in the study. [file 13071_2020_4121_MOESM1_ESM.docx]

**Additional file 1: Table S1.** Sequence information for primers used in the study.

__________________________________________________________________________

**Gene Forward primer Reverse primer**

*TGF-β*1 ATGTGCAGGATAATTGCTGC TGGTGTTGTACAGGCTGAGG

*HSP*47 ACCGAGCCCTCTTCAGTCTT GGTGATGCCCAACATAACAAT

*TIMP-*1 CGCTAGAGCAGATCAACGA AGCAGGGCTCAGATTATGCC

*COL*3*A*l ACGCAAGGCCATGAGACTCC GCAAACAGGGCCAATGTCCA

*COL*1*A*l ACTGGCAACCTCAAGAAGTCCC AAGTTCCGGTGTGACTCGTGC

*α-SMA* CTATGCTCTGCCTCATGCCA CTCACGCTCAGCAGTAGTCA

*IL*-13 GCAATGCCATCTACAGGA AGTGGGCTACTTCGATTTT

*IL*-17 TCAGACTACCTCAACCGTTCC GGTGGTCCAGCTTTCCCT

*IL*-6 GTGAAGACATACAGGGCTAAG CAGTCCCAAGAAGGCAAC

*CTGF* GCTAAGACCTGTGGAATGGGC CTCAAAGATGTCATTGCCCCC

*β-actin* ACGCAGCTCAAGTAACAGTCC CCACCATGTACCCAGGCATT

_________________________________________________________________________________
